# Beyond the Status Quo: Particulate Matter Standards in Oral Dosage Forms

**DOI:** 10.3390/jox16040151

**Published:** 2026-08-15

**Authors:** Gourav Pandey, Hitesh Chavda

**Affiliations:** 1 Takeda Pharmaceuticals, Boston, MA 01801, USA; gourav.pandey@takeda.com; 2 Independent Researcher, Gandhinagar 382010, India

**Keywords:** particulate contamination, oral dosage forms, particulate matter, analytical spectroscopy, decision pathway model, regulatory standards, regulatory harmonization

## Abstract

Particulate contamination in oral dosage forms poses significant challenges to product integrity and patient trust, yet these formulations lack the well-defined regulatory limits established for injectables. This review comprehensively analyses existing literature, regulatory guidelines, and analytical techniques to identify primary contamination sources, assess regulatory gaps, and propose science-based mitigation strategies. Key contamination vectors include raw materials, manufacturing equipment, facilities, packaging, personnel activities, utilities, processing aids, cleaning residues, and process design deficiencies. While solid oral dosage forms can encapsulate particulates, liquid formulations present a heightened ingestion risk. To combat these vulnerabilities, advanced analytical techniques—such as Fourier transform infrared spectroscopy, Raman spectroscopy, scanning electron microscopy with energy-dispersive X-ray spectroscopy, and X-ray fluorescence—are evaluated alongside emerging artificial intelligence-driven detection and real-time monitoring systems. Because current regulations inadequately address particulate matter in oral dosage forms, this paper introduces a structured decision pathway model to enhance contamination management. Ultimately, regulatory harmonization is essential for patient safety, and future research must focus on refining detection methodologies, risk-based strategies and establishing scientifically justified particulate thresholds.

## 1. Introduction

The establishment of standards for particulate matter in oral dosage forms is essential to ensure pharmaceutical product quality, safety, and therapeutic performance. Particulate matter, including dust, fibers, and foreign particles, poses significant health risks when ingested, potentially leading to adverse effects. Vulnerable populations, especially children, face increased risks due to their smaller body size and developing immune systems. To minimize these risks, robust testing methodologies, scientifically justified particle limits, comprehensive risk assessments, ethical manufacturing practices, and effective post-market surveillance are necessary. Maintaining pharmaceutical quality therefore requires a thorough understanding and strict control of particulate contamination throughout the product lifecycle [1].

The pharmaceutical industry faces challenges due to a lack of globally harmonized regulations for particulate matter in oral dosage forms [2]. Although established standards exist for injectable products [3,4,5], herbal medicines [6], and food products [7], specific regulatory guidance for oral pharmaceutical products remains limited, resulting in uncertainty regarding acceptable contamination thresholds. Greater collaboration among regulatory agencies, including the U.S. Food and Drug Administration (FDA), European Medicines Agency (EMA), and Pharmaceuticals and Medical Devices Agency (PMDA), would facilitate the development of harmonized, science-based, risk-based approaches for particulate assessment and standardized acceptance criteria. Such alignment could improve regulatory consistency, support evidence-based decision-making, and reduce unnecessary batch rejections while maintaining product quality and patient safety.

Particulate contamination during manufacturing can lead to product recalls, safety concerns, financial losses, and reputational damage. Classification systems and guidelines from industry organizations and regulatory agencies assist manufacturers in identifying contamination sources, performing risk evaluations, and implementing corrective and preventive measures. Effective collaboration between manufacturers and regulatory agencies, together with adherence to good manufacturing practices (GMP), is critical for strengthening contamination control and ensuring consistent product quality.

Effective particulate control strategies require detailed risk assessments addressing both acute and long-term exposure concerns. These assessments should examine the relationship between particle size, composition, and concentration, particularly in pediatric populations with underdeveloped gastrointestinal (GI) tracts and immune systems. Age-specific risk assessments are necessary to address the vulnerabilities of all patient groups. Additionally, the route of administration influences the risk profile, with oral dosage forms affecting drug dissolution, bioavailability, and GI mucosa, necessitating a science-based understanding of how particulates interact with the human body.

Analyzing particulate matter requires a range of techniques, each tailored to specific material types and offering unique capabilities for characterization, population studies, and elemental analysis. Fourier-transform infrared spectroscopy (FTIR) is primarily suited for organic materials, while Raman spectroscopy effectively analyzes both organic and inorganic particles. Scanning electron microscopy (SEM)—Energy-dispersive X-ray spectroscopy (EDS) is ideal for inorganic particles like metals and minerals. Automated Raman and SEM-EDS simplify population studies, and XRF offers nondestructive inorganic identification. Inductively coupled plasma mass spectrometry (ICP-MS) excels in trace element analysis but is not routine [2]. Effective testing methodologies contribute to higher confidence in product safety and quality control.

Building upon existing regulatory and industry guidance, this review integrates current evidence on contamination sources, analytical characterization, health risk assessment, particulate baseline establishment, contamination control, and regulatory considerations into a unified risk-based perspective for oral dosage forms. In addition, it presents conceptual decision pathways and integrated frameworks to support systematic particulate management and to identify future opportunities for regulatory harmonization and implementation of emerging analytical technologies.

## 2. Review Methodology

To provide a comprehensive overview of particulate matter in oral dosage forms, a structured literature search was conducted using Google Scholar, PubMed, publishers’ websites, and general Google searches. Publications from 2000 to the present were primarily considered, with earlier landmark studies included where relevant. Searches used combinations of keywords including particulate matter, foreign particles, foreign matter, oral dosage forms, tablets, capsules, liquids, contamination, analytical characterization, and technically unavoidable particles. Additional searches identified relevant regulatory guidelines, pharmacopeial requirements, and industry guidance. Literature was selected based on its scientific relevance and technical contribution to particulate identification, characterization, risk assessment, and mitigation in oral dosage forms. Key industry guidance documents—including Parenteral Drug Association (PDA) Technical Report 78 [2], Active Pharmaceutical Ingredients Committee (APIC) guidance [8], the International Pharmaceutical Excipients Council (IPEC) Technically Unavoidable Particle Profile (TUPP) Guide for Pharmaceutical Excipients [9], FDA Compliance Policy Guides (CPG) [7], and other regulatory publications —were systematically chosen based on their applicability to oral dosage forms, supply chain control, and raw material qualification.

## 3. Particulate Matter Sources and Generation Mechanisms

Particulate contamination in pharmaceutical manufacturing originates from a multifactorial interaction among raw materials, equipment and process design, manufacturing operations, facility and utility systems, packaging components, and human intervention. These contamination sources may generate viable and non-viable particulate matter through mechanisms including abrasion, corrosion, mechanical friction, thermal degradation, electrostatic attraction, material fatigue, chemical degradation, cleaning-related residues, and environmental ingress.

**Figure 1:**
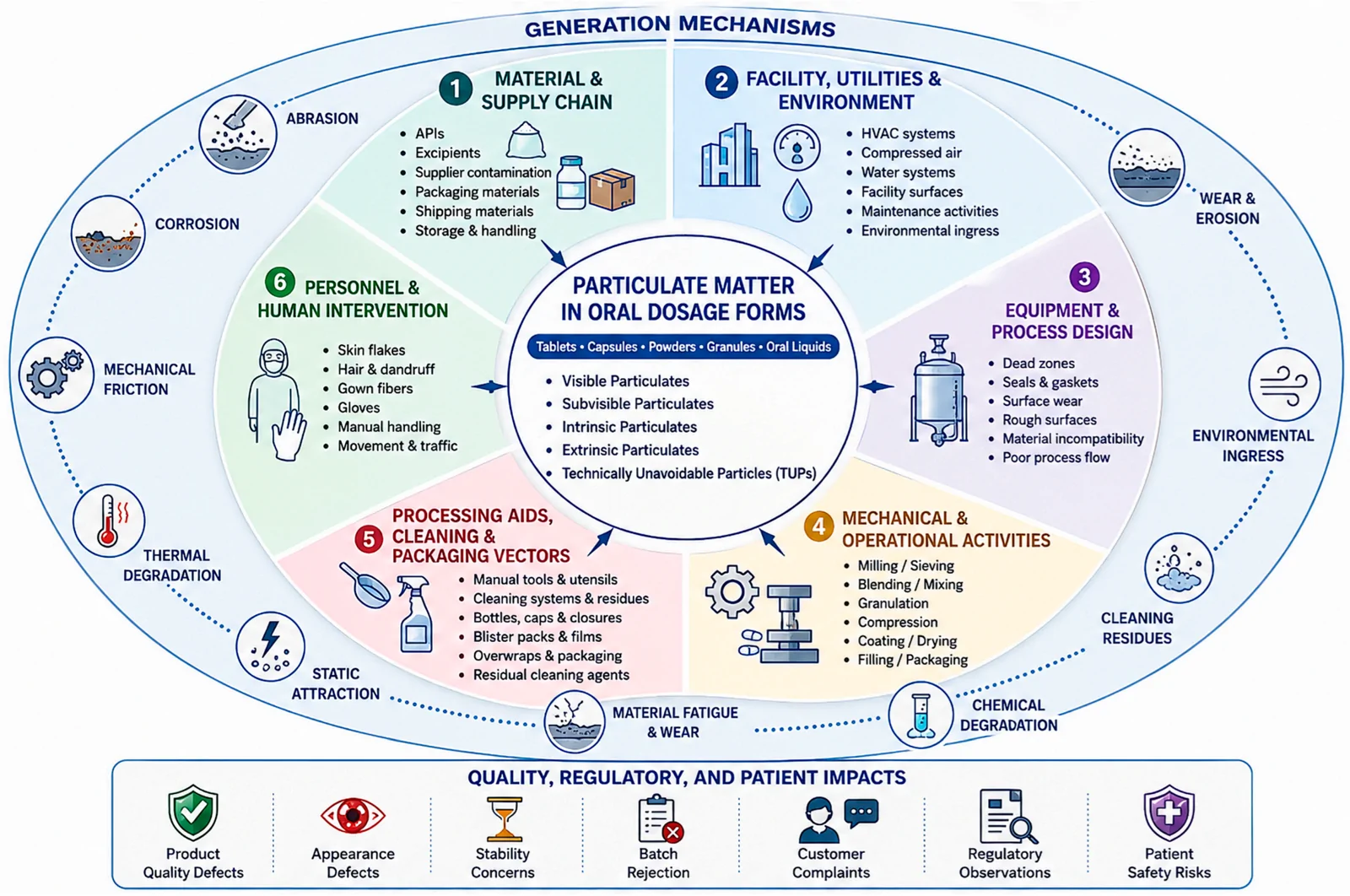
Pharmaceutical Manufacturing Ecosystem Illustrating Sources, Generation Mechanisms, and Potential Impacts of Particulate Matter in Oral Dosage Forms.

Identifying particulate sources and understanding their mechanisms of generation are essential for developing effective contamination-control and mitigation strategies. To systematically evaluate the cumulative particulate burden throughout the manufacturing lifecycle, risk factors may be broadly categorized into six interconnected domains: material and supply-chain sources, facility, utility, and environmental sources, equipment and process design-associated sources, mechanical and operational generation sources, processing aids, cleaning systems, and packaging vectors, and personnel-related variables. Collectively, these domains contribute to the overall particulate burden in oral dosage forms through multiple physical, chemical, mechanical, and environmental pathways, as illustrated in Figure 1.

### 3.1. Material and Supply Chain-Associated Sources

Raw materials provided by suppliers carry inherent risks of particulate contamination, stemming from intrinsic material properties, supplier-related contamination, chemical degradation, and product agglomeration. Additionally, risks are amplified by packaging material shedding, shipping debris, poor material handling, and environmental exposure during warehouse storage and staging. Collectively, these supply chain and handling factors contribute to particulate accumulation, presenting significant contamination risks within pharmaceutical manufacturing environments (Table 1).

### 3.2. Facility, Utility, and Environmental Sources

Facility infrastructure, utility systems, and environmental conditions continuously influence particulate burden throughout pharmaceutical manufacturing operations. Deteriorating surfaces, airborne ingress, maintenance activities, contaminated utilities, and airflow disturbances may significantly increase airborne and surface-associated particulate contamination within controlled manufacturing environments (Table 2).

### 3.3. Equipment and Process Design-Associated Sources

Poorly designed process layouts and equipment configurations may generate particulate contamination through dead zones, inadequate sealing, excessive frictional interfaces, incompatible construction materials, and difficult-to-clean surfaces. Structural deficiencies and unoptimized process flow may promote residue accumulation, product attrition, and cross-contamination risks (Table 3).

### 3.4. Mechanical and Operational Generation Sources

Mechanical operations and equipment interactions represent major contributors to particulate generation during pharmaceutical manufacturing. Friction, abrasion, vibration, corrosion, thermal stress, and equipment wear occurring during blending, granulation, compression, transfer, and packaging operations may release metallic, polymeric, and powder-derived particulates into the manufacturing environment. Representative sources associated with active manufacturing operations and equipment interactions are summarized in Table 4.

### 3.5. Processing Aids, Cleaning Systems, and Packaging Vectors

Processing aids, cleaning systems, and packaging components may serve as direct vectors for foreign particulate introduction. Improperly maintained tools, residual cleaning agents, degraded packaging materials, and incompatible contact surfaces may generate fibers, polymer fragments, residues, and other contaminants capable of compromising product quality and process integrity. Major contributors associated with processing aids, sanitation systems, and packaging interactions are presented in Table 5.

### 3.6. Personnel and Human Intervention Variables

Personnel activities remain among the most dynamic contributors to particulate contamination in pharmaceutical manufacturing environments. Human shedding, gowning deficiencies, manual interventions, and personnel movement may significantly increase airborne and surface-associated particulate burden within controlled manufacturing areas. Representative personnel-associated contamination vectors are summarized in Table 6.

## 4. Particulate Matter Risks

The presence of particulate matter in oral dosage forms poses varying risks depending on the formulation. Solid forms, such as tablets and capsules, may embed particulates within their matrix. While this reduces immediate harm, it still poses risks of altered dissolution, mechanical defects, or GI irritation. Although packaging rarely contributes to this issue, manufacturing processes remain a primary source of contamination.

In contrast, liquid formulations, including solutions and suspensions, present a higher risk, especially for vulnerable populations. Suspended particulates in liquids are directly ingested, increasing the potential for adverse effects, including organ deposition, irritation, or chemical interactions. Table 7 outlines the risks of particulate matter in oral dosage forms.

## 5. Particulate Matter Mitigation Strategies

Effective mitigation of particulate contamination in pharmaceutical manufacturing requires a proactive approach that targets all potential sources. Implementing robust supplier qualification programs, controlled material handling, and in-process filtration can significantly reduce contamination risks. Facility and equipment design should prioritize non-shedding materials, airlocks, and proper pressurization to minimize environmental particulates. Regular maintenance, inspections, and component replacements prevent particulate generation from equipment wear. Cleaning procedures must use validated low-residue agents, while stringent gowning and hygiene protocols limit human-related contamination. Additionally, utilities such as purified water and compressed air must be monitored and maintained with HEPA filtration to prevent particulate introduction. By integrating these strategies into manufacturing operations, pharmaceutical companies can ensure product integrity, regulatory compliance, and patient safety. Table 8 presents the comprehensive mitigation strategies for particulate matter in pharmaceuticals.

## 6. Particulate Baselines

Particulate monitoring in oral dosage form manufacturing is essential for ensuring product quality, patient safety, and regulatory compliance. Establishing a particulate baseline enables differentiation between inherent particulates and contamination-related events. The process begins with raw material assessment, where APIs and excipients are evaluated for inherent particulates, supplier specifications including technically unavoidable particles data and material quality attributes are reviewed, and incoming materials are screened to detect excessive particulate contamination before processing [9,20]. In-process monitoring involves filtration or screening where applicable, visual inspections during processing, and batch-to-batch trend analysis to ensure particulate levels remain within acceptable limits. Packaging materials represent another potential source of particulate contamination; therefore, bottles, caps, liners, blister packs, and sachets should be evaluated for particulate shedding, while packaging cleanliness, integrity, and supplier quality are verified through appropriate inspection and qualification activities. Finished products are assessed for visible and subvisible particulate matter, as applicable, and the results are compared against established internal limits and historical baselines. When deviations are identified at any stage, corrective actions such as supplier investigations, material rejection or reprocessing, packaging modifications, and process improvements are implemented. Maintaining a particulate library facilitates trend analysis, baseline refinement, and continuous improvement of contamination-control strategies [2]. This proactive and risk-based approach enhances process control, supports regulatory compliance, and minimizes particulate contamination in oral dosage forms.

**Figure 2:**
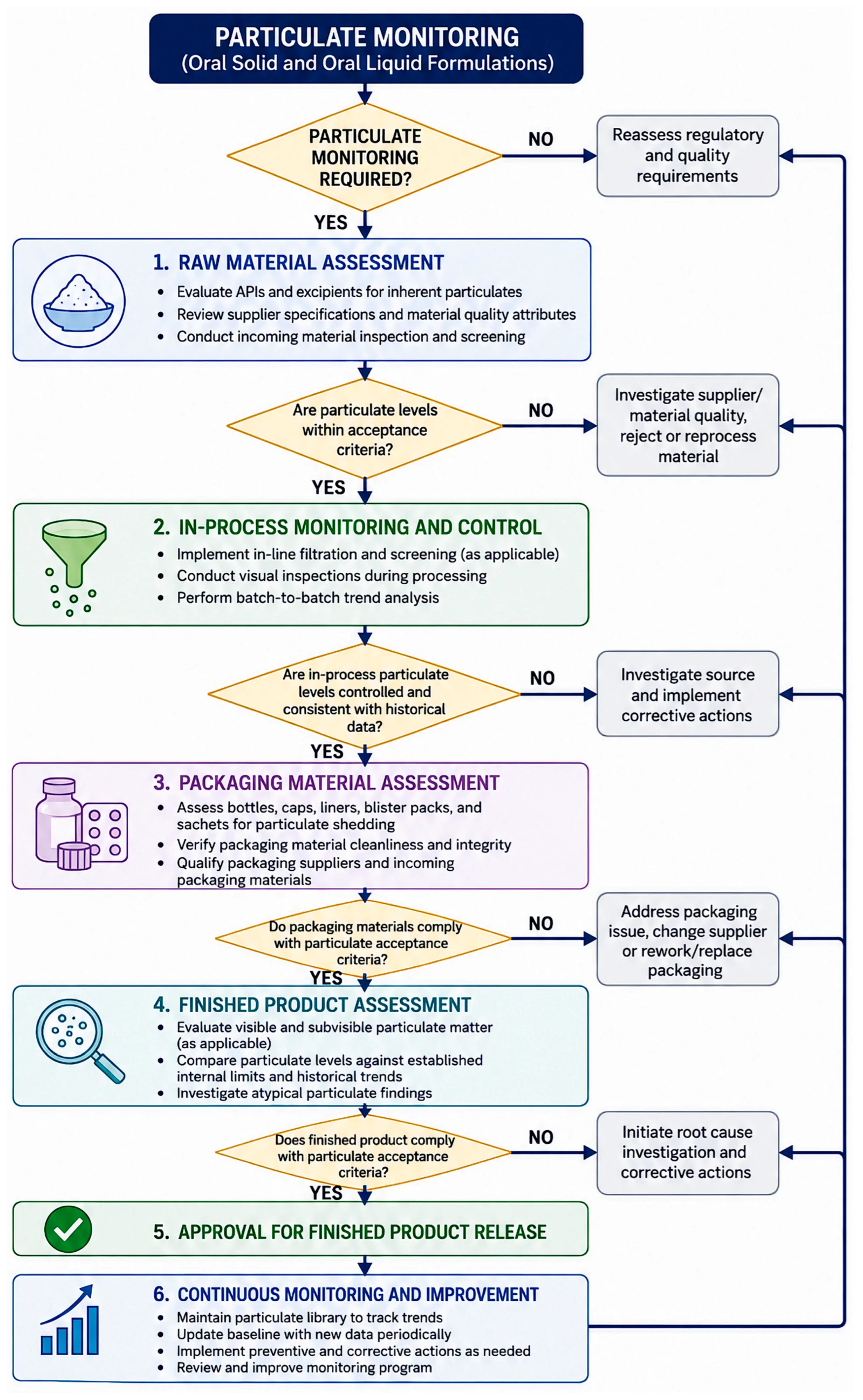
Decision Tree for Particulate Monitoring and Control in Oral Dosage Forms.

The decision tree presented in Figure 2 is intended as a conceptual framework to guide risk-based particulate monitoring and corrective actions throughout the product lifecycle. In modern high-speed manufacturing environments, its implementation should be supported by validated in-line monitoring technologies, automation, and statistical trend analysis to facilitate timely decision-making while minimizing process variability. As these technologies continue to advance, the framework can be integrated into digital manufacturing and pharmaceutical quality systems to support continuous process verification and quality improvement.

## 7. Particulate Matter Analysis

Analytical characterization plays a central role in particulate matter investigations by enabling the identification, classification, and source attribution of contaminants encountered during pharmaceutical manufacturing. Effective particulate analysis requires a systematic approach that combines morphological, chemical, and elemental characterization techniques to determine the nature and origin of contaminants. Because particulate matter may consist of organic compounds, inorganic materials, metallic fragments, polymers, excipient residues, or complex mixtures thereof, no single analytical technique is universally applicable. Consequently, a multi-technique strategy is often necessary to achieve comprehensive characterization and support root cause investigations.

### 7.1. Analytical Characterization Techniques

The analytical investigation of particulate matter typically begins with visual and microscopic examination. Optical microscopy and stereomicroscopy provide preliminary information regarding particle size, shape, color, texture, and distribution, allowing investigators to classify particulates and select appropriate analytical techniques for further characterization [21,22]. Although these methods provide valuable morphological information, they are generally insufficient for definitive chemical identification.

Spectroscopic techniques are widely employed for chemical characterization of particulate contaminants. FTIR remains one of the most commonly used methods for identifying organic materials due to its rapid, nondestructive nature and extensive spectral libraries [23,24]. FTIR is particularly effective for polymers, excipients, lubricants, and packaging-related contaminants. However, its applicability may be limited for very small particles and inorganic materials. Recent advances in micro-FTIR imaging have enhanced the capability to characterize smaller particles and heterogeneous samples with improved spatial resolution.

Raman spectroscopy provides complementary analytical capabilities and offers significant advantages for the characterization of both organic and inorganic particulates [25,26]. The technique can analyze particles at the micrometer scale with minimal sample preparation and is particularly valuable for identifying pigments, minerals, APIs, and polymeric materials. Raman mapping and automated Raman particle analysis have further expanded its application in contamination investigations. Nevertheless, fluorescence interference and complex spectral backgrounds may complicate interpretation in certain samples.

For high-resolution morphological evaluation and elemental characterization, SEM coupled with EDS is widely regarded as one of the most powerful analytical approaches [27,28,29]. SEM enables detailed visualization of particle surface characteristics, morphology, and structural features, while EDS provides qualitative and semi-quantitative elemental composition data. These techniques are particularly useful for identifying metallic particles, mineral contaminants, corrosion products, and equipment-derived debris. However, they provide limited information regarding the molecular structure of organic materials and are therefore often used in conjunction with spectroscopic methods.

Elemental analysis techniques such as XRF spectroscopy and ICP-MS further support particulate investigations. XRF offers rapid, nondestructive identification of inorganic elements and is particularly useful for metallic and mineral contaminants. ICP-MS provides highly sensitive trace elemental analysis and is frequently utilized to evaluate bulk contamination trends, although its destructive nature limits its applicability for individual particle characterization [30,31,32,33,34].

Emerging technologies continue to enhance particulate investigations within the pharmaceutical industry. Laser-induced breakdown spectroscopy, hyperspectral imaging (HSI), automated particle analysis platforms, and integrated spectroscopic-imaging systems enable rapid characterization of large particle populations while improving analytical efficiency and data quality [35,36,37,38]. These technologies facilitate more comprehensive contamination assessments and support increasingly complex manufacturing environments.

The selection of an analytical technique depends on several factors, including particle size, morphology, material composition, sample availability, and investigation objectives. In practice, combining complementary analytical methods frequently provides the highest confidence in particulate identification and source attribution [39,40].

### 7.2. Retrieval and Isolation of Particulate Contaminants

Accurate particulate characterization depends on the effective retrieval and isolation of contaminants while preserving their physical and chemical integrity. Improper sampling techniques can introduce artefacts, alter particle morphology, or result in secondary contamination, thereby compromising analytical results.

Particulate retrieval strategies vary depending on the nature of the product, the location of contamination, and the physical characteristics of the particle. Common approaches include manual micro-manipulation under stereomicroscopic observation, membrane filtration, adhesive sampling, and micro-extraction techniques for oily or semi-solid contaminants [41,42]. Polycarbonate membrane filters help isolate particles from solutions due to their smooth surface, aiding observation and collection. Membrane filtration is particularly useful for isolating particulate matter from liquid samples, whereas direct particle collection is often employed for solid dosage forms and packaging components.

Following retrieval, particles are transferred to appropriate analytical substrates compatible with subsequent characterization techniques such as FTIR, Raman spectroscopy, SEM-EDS, or XRF analysis. Throughout the sampling process, contamination control measures are essential to preserve sample integrity. Investigations should therefore be conducted under controlled environmental conditions using appropriate cleanroom practices, laminar airflow protection, and validated handling procedures.

A systematic and contamination-controlled retrieval process significantly improves analytical reliability and supports accurate source determination during particulate investigations.

### 7.3. Interpretation of Analytical Data and Source Determination

The successful resolution of particulate contamination events depends not only on analytical characterization but also on the accurate interpretation of analytical data within the broader context of pharmaceutical manufacturing operations. Source attribution requires the integration of morphological, chemical, elemental, and operational information to establish a scientifically justified contamination pathway.

Analytical data generated from FTIR, Raman spectroscopy, SEM-EDS, XRF, ICP-MS, and other characterization techniques provide complementary information regarding particle identity and composition. However, pharmaceutical particulates frequently consist of complex mixtures containing APIs, excipients, processing residues, lubricants, packaging materials, environmental contaminants, or equipment-derived particles. Consequently, spectral and elemental signatures may exhibit overlapping characteristics that complicate interpretation [43,44,45].

While commercial spectral databases provide valuable initial identification capabilities, database matching alone is often insufficient for definitive source determination. Expert interpretation remains essential for resolving ambiguous results, differentiating mixed-material spectra, and evaluating analytical findings within the context of the manufacturing process. Correlation of analytical data with raw material specifications, equipment construction materials, maintenance records, environmental monitoring data, cleaning validation records, and production history can substantially improve investigation outcomes.

The increasing adoption of digital particulate libraries and integrated analytical databases has enhanced contamination investigations by enabling rapid comparison of unknown particles with historical contamination events and reference materials [46,47]. These systems facilitate trend analysis, recurring contamination identification, and knowledge management across manufacturing sites.

A structured source attribution process typically involves particle characterization, database comparison, process correlation, hypothesis development, and confirmatory investigation. For example, metallic particulates may be linked to equipment wear through elemental composition matching and maintenance records, whereas polymeric particles may be traced to packaging components, gaskets, filters, or transfer systems. A systematic workflow using complementary analytical techniques, including light microscopy, SEM-EDX, FTIR, and time-of-flight secondary ion mass spectrometry, has demonstrated the value of integrating morphological, elemental, and chemical characterization for identifying diverse contaminants, including metallic, polymeric, glass, rubber, and pharmaceutical cross-contamination in solid dosage forms, thereby facilitating source attribution and root cause investigations [48]. Similarly, a recent study investigating microplastic contamination in oral pharmaceutical formulations combined Laser Direct Infrared Spectroscopy, pyrolysis gas chromatography–mass spectrometry, and SEM to provide complementary chemical, quantitative, and morphological characterization of polymeric particulates [49]. Although the latter study did not evaluate packaging materials separately and therefore could not conclusively distinguish manufacturing-related contamination from packaging-derived particles, it further demonstrated the value of integrating multiple analytical techniques for comprehensive particulate characterization in complex pharmaceutical matrices. The integration of analytical evidence with operational data enables investigators to identify root causes with greater confidence and implement targeted corrective and preventive actions (CAPA).

Advances in automated data analysis, machine learning, and predictive analytics are expected to further improve particulate investigations by enhancing pattern recognition, accelerating source identification, and supporting proactive contamination control strategies. These emerging approaches align with modern pharmaceutical quality systems and contribute to the continuous improvement of product quality, patient safety, and manufacturing robustness.

## 8. Particulate Matter Medical Considerations

### 8.1. Physical Injury Risks from Particulates

Hard or sharp particulates in oral pharmaceuticals have the potential to cause mechanical injury to the oropharyngeal and GI tracts. The FDA considers objects ≥ 7 mm hazardous because they may cause lacerations, perforations, or secondary infections [7]. While smaller particulates are generally considered less injurious, they may present a greater potential risk for vulnerable populations, including infants, elderly patients, and individuals with esophageal abnormalities. Chewable formulations require careful control to minimize the possibility of dental damage from embedded particulates. Although clinically documented GI obstruction associated with particulate contamination in oral pharmaceutical products is limited, neonates may be more susceptible because of their narrower esophageal diameters.

### 8.2. Mechanical Obstruction of the Airway Due to Particulates

Liquid oral formulations have the potential to pose a risk of airway obstruction if particulates are aspirated, particularly in vulnerable populations such as infants, the elderly, or patients with dysphagia. Particulate size and morphology influence obstruction potential, with larger or irregularly shaped particles considered more likely to increase this risk. Aspiration of particulate matter may result in inflammation, bronchial irritation, or secondary infections, such as pneumonia. Improper administration techniques, including supine dosing, may further increase the likelihood of aspiration. Neonates have smaller tracheal diameters (4–5 mm) [50], which may increase their susceptibility to airway obstruction following aspiration of particulate matter. Although direct clinical evidence linking particulate contamination in oral pharmaceutical products to airway obstruction is limited, age-specific risk assessments should consider physiological differences, including airway dimensions, swallowing ability, and aspiration susceptibility, when evaluating the potential clinical impact of particulate contamination. Furthermore, clinically established particulate thresholds for oral dosage forms are currently lacking; therefore, these considerations may help inform the future development of scientifically justified, risk-based acceptance criteria for vulnerable patient populations [51,52].

### 8.3. Toxicological Assessment

Leachables from particulates may present toxicological risks depending on their chemical composition, patient exposure, and duration of exposure. Toxicological evaluation involves identifying potential contaminants, assessing estimated patient exposure levels, and comparing these data with established regulatory thresholds where applicable [53]. Patient exposure is estimated based on the maximum daily dose and compared with acceptable daily intake or permitted daily exposure (PDE) limits in accordance with the International Council for Harmonisation of Technical Requirements for Pharmaceuticals for Human Use (ICH) Q3D guideline [53]. The safety margin—calculated as the ratio of the PDE to the estimated clinical exposure—provides a risk-based assessment, with values > 1 indicating that the estimated exposure is below the toxicological threshold of concern. However, direct clinical evidence relating particulate-associated leachables in oral pharmaceutical products to adverse health outcomes remains limited. Therefore, these assessments are intended primarily to support science-based risk evaluation until additional clinical evidence becomes available. Root-cause analysis of contamination sources enables manufacturers to implement preventive measures and minimize potential toxicological hazards.

### 8.4. Health Impacts

The potential health impacts of pharmaceutical particulates depend on their size, shape, chemical composition, and frequency of exposure [54]. Larger, sharp particulates may cause localized mechanical tissue damage, while exposure to toxic contaminants, such as heavy metals or organic leachables, may present systemic health risks. Vulnerable populations, including infants, the elderly, and immunocompromised individuals, may be more susceptible to adverse effects because of physiological and clinical factors [7,55,56]. Repeated exposure to particulate-contaminated products may increase cumulative systemic exposure and has the potential to contribute to long-term toxicological concerns [57]. However, direct clinical evidence linking particulate contamination in oral pharmaceutical products to chronic adverse health outcomes remains limited. Consequently, these potential health impacts are largely inferred from general toxicological principles and related evidence and should be regarded as hypothesis-generating until supported by additional clinical and epidemiological studies. Additionally, the presence of visible particulates may undermine patient trust and reduce treatment adherence due to concerns regarding product quality and safety.

## 9. Metal Particles

Metal contamination in oral solid dosage forms, particularly tablets [58,59,60,61,62], poses significant safety and regulatory challenges for pharmaceutical manufacturers. Metal particles can appear as discrete foreign bodies or dispersed particulates within the tablet blend, with each scenario requiring tailored detection and mitigation strategies. Discrete metal particles, which are typically larger, risk causing localized mechanical injuries such as GI lacerations or perforations. Conversely, dispersed fine particles pose toxicological threats, particularly if elemental impurities such as lead (Pb) or cadmium (Cd) leach into the physiological system. Because these contaminants cannot always be fully eliminated during manufacturing, they must be rigorously evaluated against safety limits to ensure they do not compromise efficacy or exceed established toxicological thresholds [59]. The clinical risk associated with metallic contamination depends strictly on particle size, chemical composition, and morphology, emphasizing the necessity of robust detection and risk assessment methods.

Inline metal detectors positioned at critical control points, such as post-blending and pre- or post-compression stages, are highly effective in identifying macro-scale metallic contaminants [63]. For trace analysis, advanced analytical techniques like ICP-MS and XRF enable precise quantification of heavy metals, while laser ablation techniques provide spatially resolved, micro-destructive trace metal analysis [64,65,66,67]. Acceptable quality limits (AQL) are traditionally used to evaluate batch contamination levels; however, limitations in contamination homogeneity necessitate adopting a strict, risk-based approach with lowered thresholds for metallic impurities [68,69] in accordance with ICH Q3D guidelines.

Preventive strategies rely on regular equipment inspections and preventative maintenance protocols to eliminate wear-and-tear-related contamination [70], as well as integrating high-strength rare-earth magnets in transfer lines to capture upstream ferrous particles. Collaborating with raw material suppliers to enforce stringent quality standards further reduces upstream contamination risks [70,71]. When contamination is identified, isolating the affected batch and performing a comprehensive root cause analysis is critical, followed by immediate CAPA to prevent recurrence. Enhanced stratified sampling and content uniformity testing further improve detection accuracy.

In cases where contamination levels exceed regulatory thresholds, batch rejection is mandatory. This decision should be guided by toxicological data, particle characteristics, and calculated safety margins based on PDE values. Continued advancements in inline detection technology, harmonized international regulatory standards, and computational tools for blending optimization will further enhance contamination control in solid oral dosage manufacturing.

## 10. Non-Metallic Particles

Non-metallic particulates, such as human hair, cellulose, synthetic fibers, and other extrinsic contaminants, present unique quality control challenges during pharmaceutical manufacturing [72,73,74]. Unlike metallic impurities, which can be readily isolated using inline inductive metal detectors, non-metallic contaminants possess low magnetic susceptibility and require specialized, high-resolution technologies to ensure product safety, critical quality attributes (CQAs), and regulatory compliance during final inline quality control.

X-ray inspection systems are widely utilized to identify dense foreign objects within finished product matrices [75,76]. These systems operate by detecting differentials in mass attenuation coefficients and material density, allowing for the reliable interception of glass fragments, stone, and high-density polymers. However, conventional X-ray systems exhibit low sensitivity when screening for low-density organic contaminants such as hair and fine fibers [77]. To detect these surface-level contaminants, optical inspection systems—leveraging advanced image recognition algorithms, high-resolution digital imaging, laser scanning, HSI, and ultraviolet (UV) fluorescence—are deployed [78,79,80,81,82,83,84,85]. While highly effective at identifying fibers and hairs on the surface of tablets or capsules, the primary limitation of optical inspection remains its inability to detect deeply embedded particulates within an opaque solid dosage matrix. Furthermore, detection efficiency is highly dependent on operational parameters such as processing line speed, ambient lighting stability, and sensor resolution settings.

HSI offers a sophisticated diagnostic alternative by combining spatial imaging with spectroscopic data to analyze the chemical composition of materials [83,86,87,88]. By differentiating particulates based on their unique near-infrared or short-wave infrared spectral fingerprints, HSI can distinguish foreign cellulose and polymeric fibers from the surrounding excipient matrix. Although HSI offers superior chemical discrimination and contaminant characterization compared with conventional optical inspection methods, its routine implementation in pharmaceutical manufacturing remains limited by high capital investment, computational demands for real-time data processing, and the requirement for product-specific calibration models. In contrast, established technologies such as X-ray and optical vision systems continue to be the preferred choice for routine in-line inspection because of their proven reliability, ease of integration into existing manufacturing lines, and regulatory acceptance. As computing capabilities, machine learning algorithms, and sensor technologies continue to advance, HSI is expected to become increasingly feasible for targeted applications and high-value products, complementing rather than replacing conventional inspection systems. Complementing these methods, laser-based surface profiling systems are highly effective at detecting micro-scale surface irregularities and contaminant particles on both reflective and non-reflective surfaces [89,90,91]. For liquid formulations, acoustic sensors that monitor sound wave interruptions caused by suspended foreign objects during product flow offer an excellent pathway for identifying fibers and particulates that disrupt the acoustic signal [92,93,94,95].

Beyond analytical instrumentation, holistic process engineering controls are crucial to mitigating contamination risks. These include multi-stage orthogonal detection arrays [96,97,98], strict cleanroom environmental controls [99,100,101], and rigorous raw material supplier screening protocols [102,103,104]. Emerging innovations, such as artificial intelligence (AI)-driven computer vision systems [105,106,107] and automated electrostatic collection devices [108,109], show promise for enhancing particle interception accuracy and operational throughput. To transition from experimental deployment to GxP-compliant routine production, these AI systems must overcome the “black-box” validation barrier inherent to deep learning architectures [110]. This is increasingly achieved by integrating Explainable AI methodologies—such as local interpretable model-agnostic explanations or feature activation maps—which visually trace the specific pixel clusters that triggered a defect classification [111]. Furthermore, compliance with GAMP 5 (Good Automated Manufacturing Practice) software category 5 requirements is maintained by establishing a hybrid software architecture: a deterministic, rule-based algorithmic framework bounds the machine learning model, ensuring that the AI functions within strict, pre-validated operational limits [112,113]. Rigorous lifecycle validation—including data integrity protections, comprehensive testing against adversarial “edge-case” artifact libraries, and robust continuous model monitoring protocols—is required to satisfy regulatory scrutiny during final product release [114]. While technical boundaries and cost constraints persist, the strategic integration of validated multi-modal detection systems, real-time monitoring, and proactive quality risk management (QRM) can strengthen the effective control of non-metallic particulates while supporting regulatory compliance.

**Figure 3:**
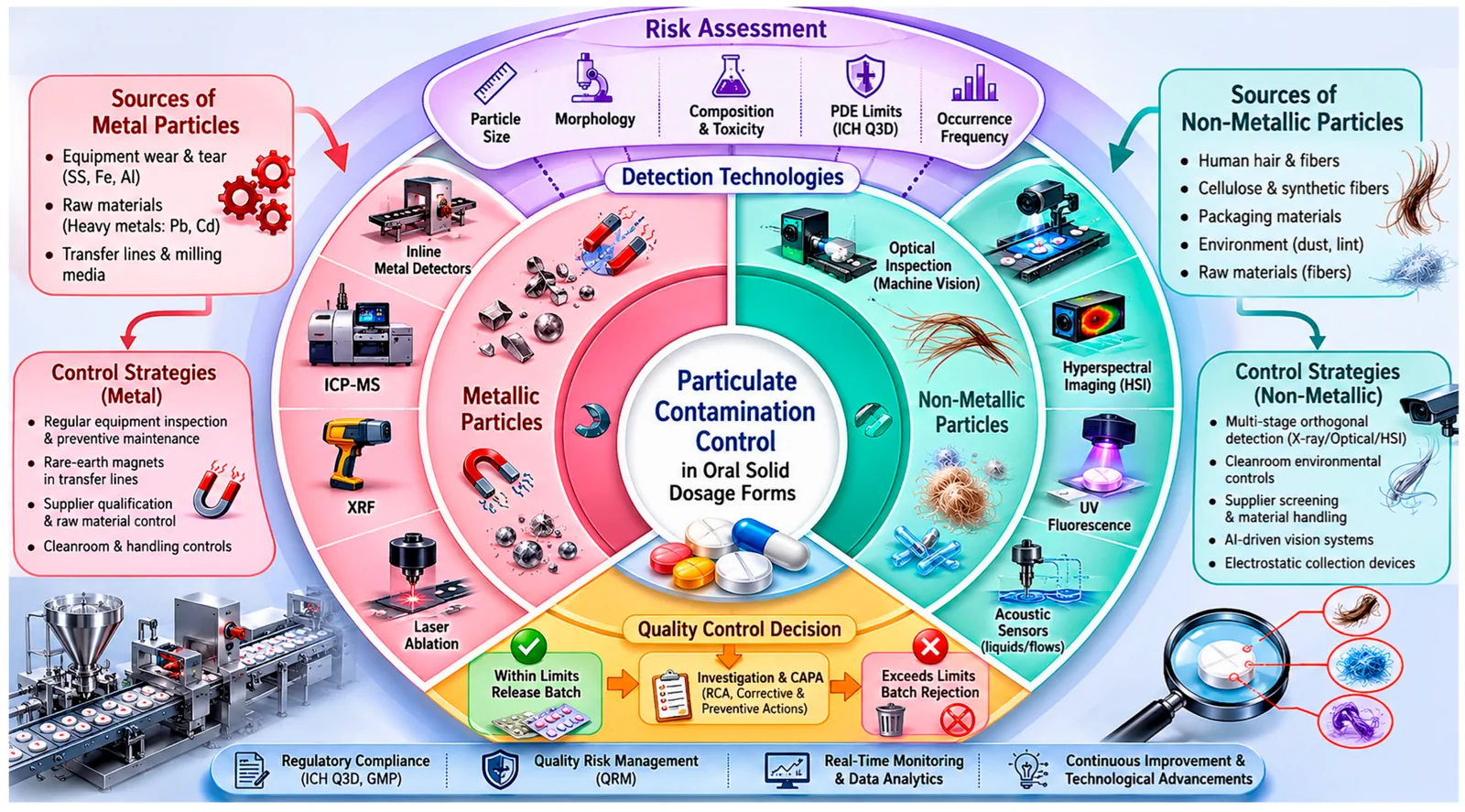
Integrated framework for detection, risk assessment, and control of metallic and non-metallic particulate contamination in oral solid dosage manufacturing.

An integrated risk-based framework for the detection, assessment, and mitigation of metallic and non-metallic particulate contamination throughout oral solid dosage manufacturing is presented in Figure 3.

## 11. Inherent Particulate Formation and Instabilities in APIs and Excipients

Unavoidable particulates and physical instabilities in APIs and excipients can significantly compromise product quality, patient safety, and regulatory compliance. These solid state anomalies can arise from raw material variability, aggressive processing conditions, or environmental storage factors, making it essential for manufacturers to map their root sources and implement proactive QRM strategies. While minor particulate generation or phase transformation is occasionally inevitable due to the intrinsic physicochemical properties of the ingredients, mitigating these risks requires an orthogonal approach combining rational material selection, optimized unit operations, and stringent inline quality controls. Table 9 summarizes the primary vulnerabilities of specific API categories to particulate growth and physical degradation during manufacturing, alongside their clinical and processing implications.

Table 10 categorizes excipient classes [115] prone to particulate anomalies, identifying their mechanistic origins and their specific impacts on final dosage form safety and performance.

## 12. Particulate Control by Manufacturers

Pharmaceutical manufacturers can significantly reduce their reliance on contract manufacturing organizations (CMOs) by proactively evaluating and controlling particulates internally. This holistic approach encompasses raw material fingerprinting, stress testing, compatibility assessments, strict environmental controls, and the deployment of sensitive analytical methods to mitigate particulate risks.

To minimize contamination, manufacturers must conduct thorough evaluations of incoming raw materials. Qualifying multiple suppliers ensures material consistency and reduces single-source dependencies. Stress testing is crucial for assessing material behavior under extreme processing conditions. For instance, thermal stress testing identifies specific temperature thresholds at which chemical degradation occurs. Similarly, mechanical stress testing simulates high-energy operations—such as compression, milling, or granulation—to evaluate material resilience and predict particulate generation stemming from brittleness or particle fracture.

Evaluating excipient–excipient and API–excipient interactions prevents chemical and physical particulate formation during formulation. Compatibility studies assess the stability of active ingredients and excipients under varied processing conditions, pinpointing degradation risks before scaling up. Furthermore, optimizing the manufacturing environment and equipment design remains vital. Implementing closed processing systems, low-shear granulators, and hygienic design principles minimizes mechanical shedding. Real-time airborne particle counters monitor cleanroom environments continuously, allowing for immediate corrective actions. In-line monitoring via Process Analytical Technology (PAT) ensures that micro-particulates are detected during active processing before they compromise final product quality.

Advanced analytical methods—such as SEM-EDS and Microscopic FTIR—are essential. These techniques differentiate between intrinsic product-related particles and extrinsic contaminants, enabling manufacturers to pinpoint root causes and evaluate impacts on product safety. Rigorous supplier qualification, regular audits, and strict material acceptance criteria further safeguard quality. Additionally, developing in-house downscaled models to simulate real-world manufacturing lines helps predict particulate generation risks, while accelerated stability studies forecast long-term particulate formation during shelf-life storage.

Manufacturers should establish clinically relevant limits for unavoidable particulates, validated through formal QRM assessments. Continuous collaboration with CMOs and vendors is essential to improve global process capabilities. Ultimately, manufacturers must advocate for standardized regulatory guidelines regarding acceptable particulate thresholds in solid oral dosage forms to minimize contamination risks globally.

**Figure 4:**
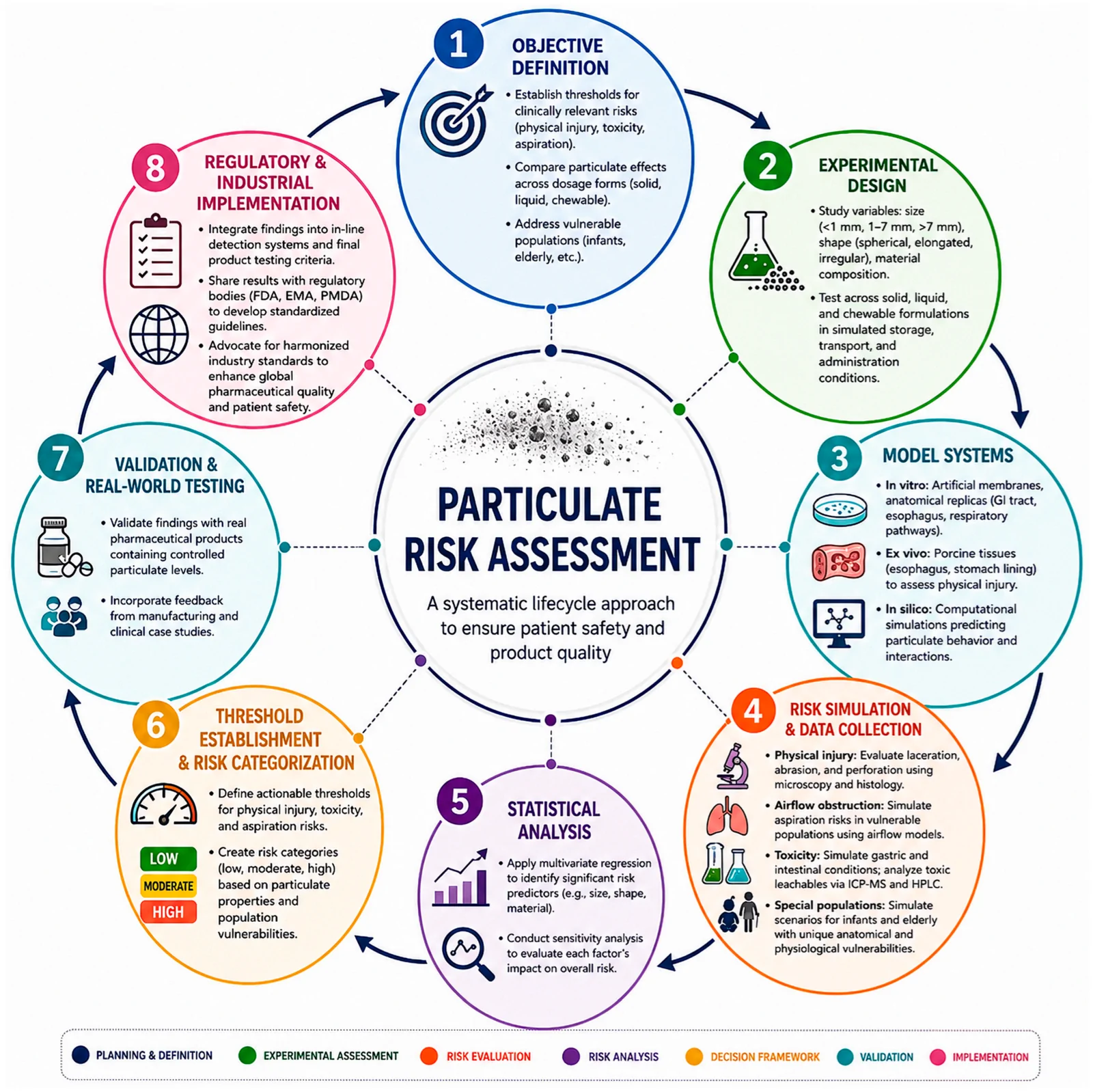
Integrated Methodological Framework for Assessing Particulate Risks in Oral Dosage Forms.

Further research is required to establish baseline thresholds for excipient- and API-specific particulates. Delineating the exact correlation between particulate size, morphology, and clinical relevance will significantly enhance modern risk assessment methodologies. Finally, developing novel detection systems for non-metallic contaminants will improve real-time inspection capabilities on the manufacturing floor. A structured methodology aimed at systematically addressing these particulate risks, balancing scientific rigor with practical manufacturing application, is illustrated in Figure 4.

## 13. Guidance Documents

### 13.1. Guidance on Handling of Insoluble Matter and Foreign Particles in APIs

The APIC Guidance on Handling Insoluble Matter and Foreign Particles in APIs provides a standardized framework for manufacturers, customers, and regulatory stakeholders to establish acceptable particle limits and investigate deviations [8]. This guidance promotes a scientific understanding of particle presence, shares engineering best practices to minimize contamination risks, offers standardized test methods with acceptance criteria, and outlines robust investigation tools for root-cause analysis. To support GMP compliance and maintain supply chain reliability, the document covers clear definitions of particles across APIs, intermediates, and raw materials. Furthermore, it outlines essential analytical controls, sampling procedures, acceptance criteria, and incident management practices—including CAPA and formal risk assessments. Integrating these standardized APIC protocols ensures robust quality control, helps differentiate between intrinsic and extrinsic particles, and maintains regulatory compliance during commercial API production [8].

**Figure 5:**
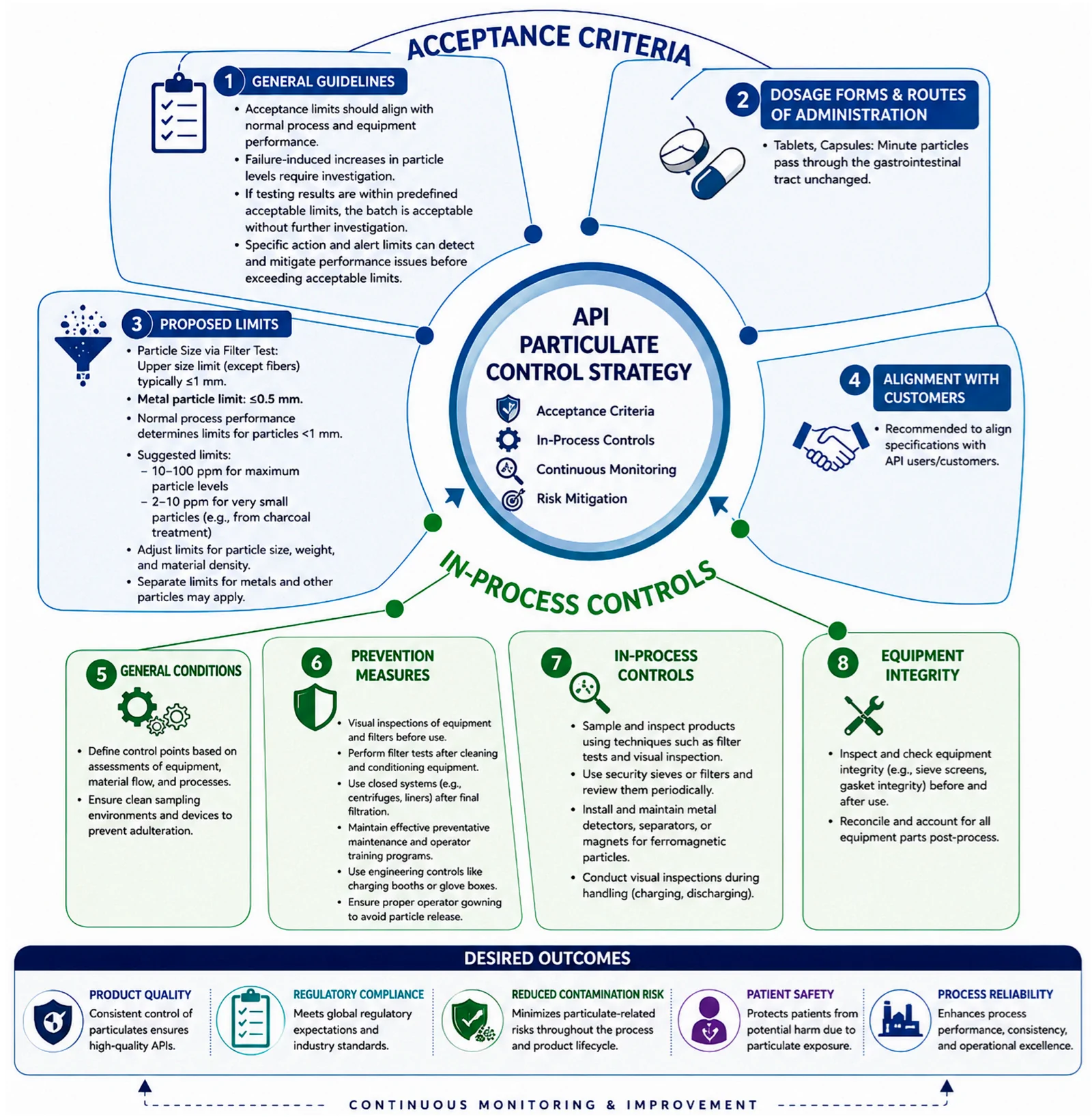
Integrated Acceptance Criteria and In-Process Control Strategy for API Particulate Management.

**Figure 6:**
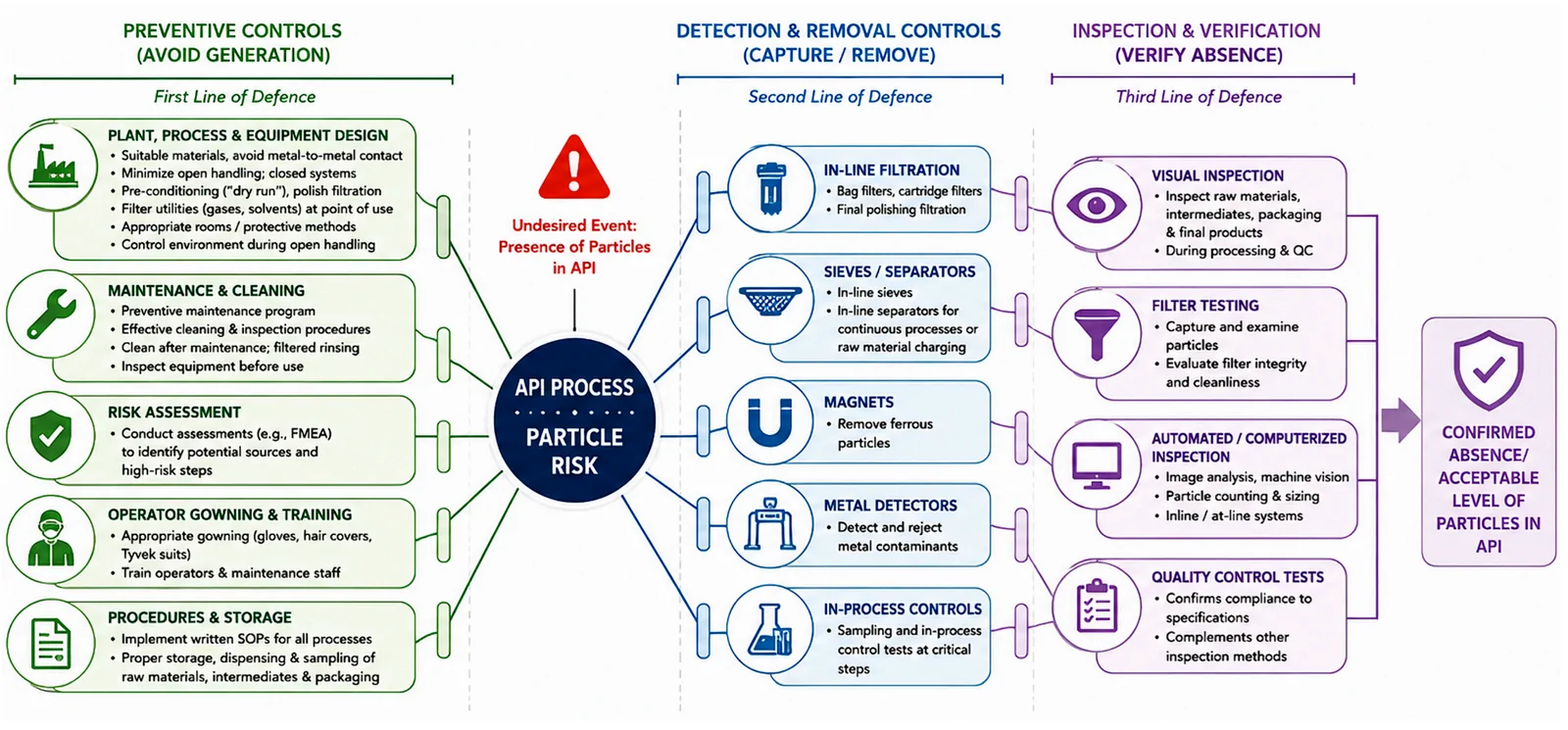
Defence-in-Depth Strategy for Particulate Control in API Manufacturing.

Figure 5 depicts a comprehensive framework for establishing particle acceptance criteria and implementing risk-based in-process controls during API manufacturing. Figure 6 outlines a multilayered particulate control strategy that combines preventive, detection, and inspection measures to reduce contamination risks across all stages of the manufacturing process.

### 13.2. Technically Unavoidable Particle Profile Guide for Pharmaceutical Excipients

A critical milestone in the modernization of excipient quality management is the IPEC TUPP Guide (Version 2, 2024) [9]. This regulatory framework standardizes how manufacturers and users differentiate inherent, process-related particles from foreign contamination. By establishing a clear baseline for visible, technically unavoidable particles, the guide provides a structured methodology for risk assessment and resource optimization during quality investigations. Manufacturers should utilize supplier-provided TUPP documentation, incoming material characterization, and historical particulate trend data to distinguish inherent particulate profiles from atypical findings during routine manufacturing. When deviations from the established baseline are identified, confirmatory analytical characterization and risk-based investigations should be performed before attributing the findings to extrinsic contamination. Crucially, the 2024 update clarifies supplier-user responsibilities and streamlines data sharing, fostering the collaborative framework necessary to maintain compliance in non-parenteral formulations while minimizing unnecessary batch rejections and supply chain disruptions.

**Figure 7:**
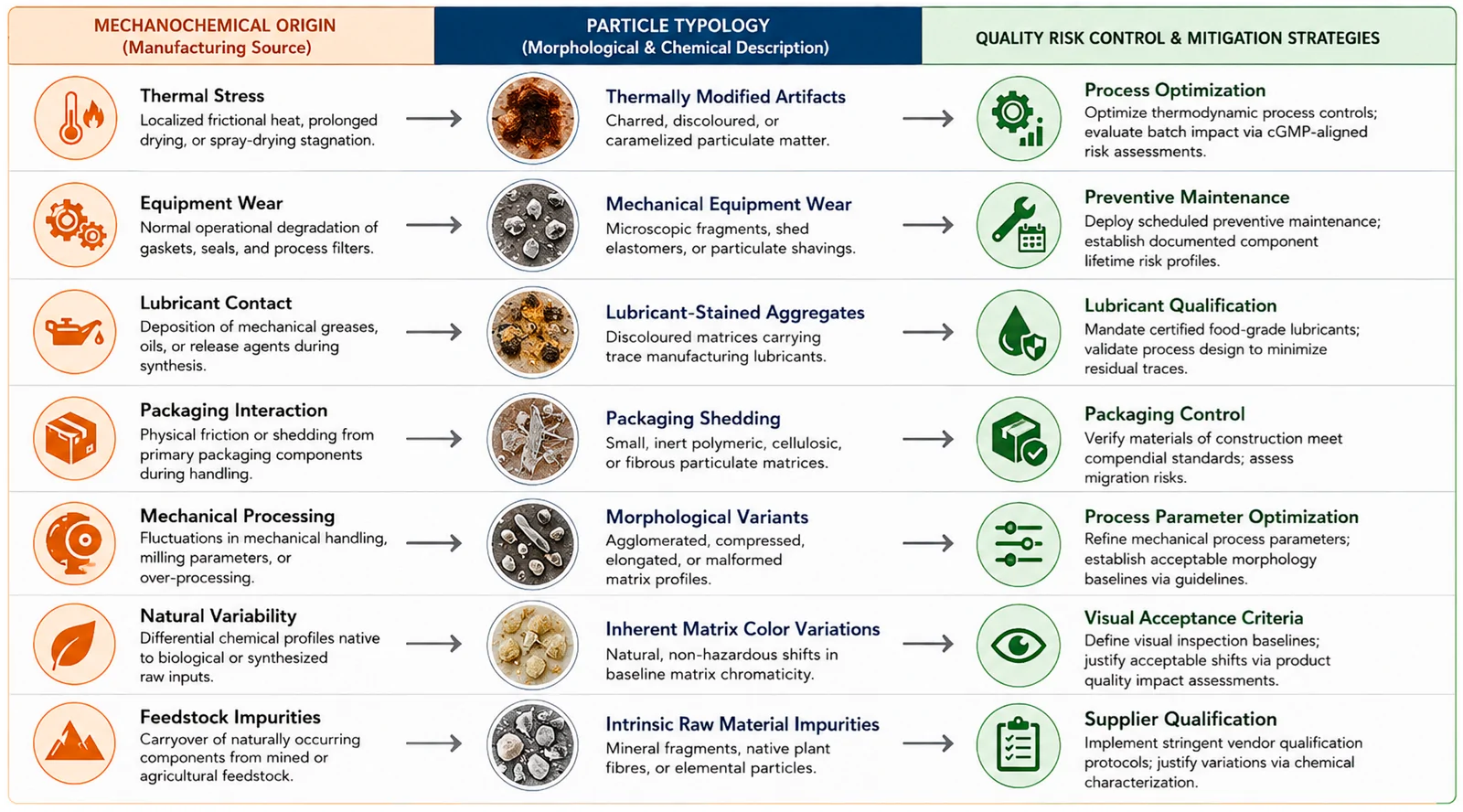
Typologies, Mechanochemical Origins, and Mitigation of Technically Unavoidable Particles in Pharmaceutical Excipients.

Figure 7 summarizes the major categories of technically unavoidable particles encountered in pharmaceutical raw materials and manufacturing processes. These particle typologies arise from thermal stress, equipment wear, lubricants, packaging components, process variability, natural material characteristics, or intrinsic impurities, together with corresponding QRM and mitigation strategies in pharmaceutical excipients.

### 13.3. Foods, Adulteration Involving Hard or Sharp Foreign Objects—Cross-Industry Perspective

To contextualize risk assessment frameworks for oral dosage forms, valuable parallels can be drawn from established food safety regulations, such as the FDA CPG Sec. 555.425 regarding hard or sharp foreign objects. In food safety matrices, objects smaller than 7 mm are statistically shown to rarely cause severe internal injury, except within high-risk patient populations such as infants, pediatric patients, or the elderly. Conversely, natural product components—such as micro-fragments of bone or shells—are generally classified as technically unavoidable unless the product is specifically labeled otherwise.

The FDA enforces strict defect action levels to govern these unavoidable physical anomalies. Under this framework, a product is legally deemed adulterated if it contains foreign objects measuring between 7 mm and 25 mm in length, provided the product is in a ready-to-eat or minimally processed form. While these dimensions primarily govern dietary matrices, understanding the FDA’s health hazard evaluation criteria—which weigh processing steps, mechanical breakdown during ingestion, and intended patient demographics—provides a robust scientific foundation for establishing safety baselines and CQAs for oral dosage forms [7].

## 14. Conclusions

The issue of particulate matter in oral dosage forms presents a significant yet largely under addressed challenge within the pharmaceutical industry. Despite some valuable insights offered by the PDA, there remains a critical lack of comprehensive and standardized regulatory guidance on particulate contamination in oral dosage forms. This regulatory void highlights the urgent need for more robust, science-based frameworks to address the sources, detection, and mitigation of particulates, ensuring the safety and efficacy of pharmaceutical products. While advancements in detection technologies and mitigation strategies are being explored, the absence of clear guidelines on acceptable particulate levels, particularly in relation to different drug formulations, remains a considerable barrier. Furthermore, the current reliance on industry-specific surveys, such as those conducted by the PDA, underscores the necessity for regulatory bodies to take a more proactive role in establishing internationally harmonized standards for particulate matter. This includes the development of clear thresholds for particulate risk, the adoption of rigorous testing protocols, and the integration of health risk assessments that consider the diverse clinical implications of particulates. The need for such guidance is further amplified by the variability in particulate sources, from raw materials to manufacturing processes, and the potential risks they pose to patient safety, especially in vulnerable populations. Regulatory bodies must acknowledge this issue as a critical area of concern, necessitating targeted efforts to refine and standardize the approach to particulate matter in pharmaceutical products. Only through these concerted regulatory efforts can the industry ensure that oral dosage forms meet the highest standards of quality, safety, and patient care.

By integrating current knowledge into conceptual risk-based frameworks and decision pathways, this review provides a structured approach for systematic risk assessment, root cause investigations, and particulate management throughout the pharmaceutical product lifecycle. Although these frameworks require further validation through industrial implementation and regulatory experience, they may support future refinement of particulate management strategies, regulatory harmonization, and the integration of emerging analytical and digital technologies.

Although regulatory guidance for particulate matter in oral pharmaceutical dosage forms remains limited, existing scientific knowledge provides a foundation for future harmonization. A risk-based framework for establishing particulate acceptance criteria could consider particle size, morphology, composition, quantity, patient population, dosage form, route of administration, and toxicological significance. Such an approach may draw upon principles from FDA CPG Sec. 555.425 for physical hazards and toxicological risk assessment frameworks, including ICH Q3D, while remaining specific to pharmaceutical quality and patient safety. Current industry guidance, particularly PDA Technical Report 78, together with recommendations from APIC and the IPEC TUPP Guide, may provide a scientific basis for the future development of pharmacopeial chapters or regulatory guidance dedicated to oral pharmaceutical dosage forms. Global harmonization could be facilitated through collaborative initiatives involving regulatory agencies, pharmacopeial organizations (e.g., USP, European Pharmacopoeia, and the Japanese Pharmacopoeia), and international organizations such as ICH to establish science-based definitions, standardized analytical methodologies, reporting practices, and risk-based acceptance criteria. Although these recommendations are conceptual and require further scientific validation and regulatory consensus, they provide a practical framework to support the future standardization of particulate matter control in oral pharmaceutical products.

Regulatory non-compliance due to particulate matter can lead to warnings, import bans, and product seizures. The presence of particulates often necessitates costly recalls, causing financial losses and supply chain disruptions. Consumer trust may decline, damaging brand reputation and market share. Particulate matter can indicate broader manufacturing deficiencies, such as poor equipment maintenance and ineffective environmental controls, leading to batch rejections and production failures. These challenges delay product availability and require extensive investigations. Heightened regulatory scrutiny results in frequent inspections, stricter compliance measures, and operational burdens. Persistent issues with particulate matter can lead to loss of manufacturing licenses, exclusion from key markets, and reduced profitability, threatening long-term business sustainability.

## Figures and Tables

**Table 1:** Material and Supply Chain-Associated Sources.

Risk	Source	Mechanism of Generation	Examples
**Raw Materials**	Intrinsic Properties	Agglomeration, static charge, or size inconsistencies	Fine powder cake, localized agglomerates
	Chemical Reactivity	Material degradation or chemical reactions under unsuitable conditions	Crystalline precipitates, degradation by-products
	Dispensing & Sampling	Improper handling practices or use of non-dedicated tools	Tool residues, skin flakes, foreign particles
	Supplier Processes	Inadequate filtration, material handling, or cross-contamination during manufacturing	Residual impurities, processing by-products
	Equipment Wear	Abrasion, corrosion, or degradation of processing equipment components	Metal shavings, polymer fragments, gasket particles
	Environmental Factors	Ambient airborne contaminants settling during storage or staging	Atmospheric dust, moisture-induced particles
	Packaging Materials	Shedding of fibers or particulate matter during handling, storage, or transportation	Bag fibers, carton particles, plastic fragments
	Shipping Materials	Particulates from pallets, sacks, liners, or transport containers	Dust, wood splinters, packaging debris

**Table 2:** Facility, Utility, and Environmental Sources.

Risk	Source	Mechanism of Generation	Examples
**Facility & Infrastructure**	Walls, Ceilings, Floors	Surface degradation, mechanical impact, or structural wear on architectural coatings	Epoxy/paint chips, concrete dust, plaster debris, sealant flakes
	Doors and Openings	Differential pressure drops and ambient drafts entering via unsealed perimeters during transit	External environmental dust, pollen, outdoor particulates
	Unsealed Junctions	Mechanical shifting or erosion of unsealed material edges and construction joints	Insulation fibers, construction debris, dry-wall dust
	Lighting Fixtures	Thermal stress or physical degradation of unsealed or non-flush overhead units	Glass shards, brittle plastic fragments, housing paint flakes
	Maintenance Activities	Localized drilling, welding, grinding, or component replacement during repair, facility modification, or maintenance work	Metal shavings, tool debris, aerosolized lubricants, weld slag
	Waste Disposal Areas	Turbulent airflow or poor containment during the movement and segregation of waste	Airborne refuse dust, localized microbial blooms, waste particles
**Utilities**	Heating, Ventilation, and Air Conditioning (HVAC) System & Ductwork	High-Efficiency Particulate Air (HEPA) filter seal bypass, gasket degradation, or desiccation flake-off inside supply duct walls	Atmospheric dust, synthetic fibers, microbial contaminants
	Vacuum Extraction Systems	Differential pressure spikes driving mechanical powder breakthrough or dust-collector filter matrix rupture	Recovered powder fines, synthetic filter media fibers
	Compressed Air Lines	Desiccant bed attrition, compressor seal breakdown, or pipeline moisture corrosion	Hydrocarbon aerosol condensates, desiccant dust, ferric oxide scale
	Inert Process Gases (Nitrogen, Oxygen)	Cylinder hookup friction or purifier bed degradation during active purging/blanketing	Fine particulate metallic debris, micro-droplets
	Purified Water Loops	Turbulent velocity erosion, bio-film shearing, or rouging within non-passivated distribution loops	Sloughed bio-film fragments, precipitated mineral scales, rouging dust.
	Clean/Pure Steam Supply	High-velocity thermal shock peeling away pipe surface scales or boiler feed carryover	Magnetite particulates, non-volatile chemical residues
	Equipment Cooling Jackets	Thermal cycling stress and localized pinhole leaks in heat exchangers introducing cooling media into process zones	Precipitated mineral scales, galvanic corrosion flakes, microbial bio-mass

**Table 3:** Equipment and Process Design-Associated Sources.

Risk	Source	Mechanism of Generation	Examples
**Process Design**	Open Processes	Direct exposure of exposed product streams to ambient room air during transfer or sampling	Room dust, airborne fibers, operator shedding
	Powder Transfer Systems	High-velocity friction, seal wear, or line venting during pneumatic/mechanical conveyance	Concentrated dust clouds, escaped material fines
	Static Build-up	Triboelectric charging of non-conductive powders causing wall adhesion or sudden discharge	Powder clusters, static-attracted debris
	High-Shear Operations	Severe mechanical stress, particle attrition, or blade friction during mixing or milling	Fractured powder fines, eroded blade fragments
	Unoptimized Process Flow	Poor segregation of clean/dirty zones or overlapping batch timelines in the same area	Cross-batch residues, inter-product contaminants
	Thermal Processing	Localized overheating, product baking, or sublimation on hot surfaces/heating elements	Charred residues, vapor condensates, ash
**Improperly Designed Equipment**	Complex Equipment Layouts	Difficult-to-clean areas, fluid dynamic dead zones causing material stagnation and cleaning failure	Residual formulation powders, crystallized sanitization residues
	Rough or Uneven Micro-Surfaces	High surface roughness accelerating mechanical product abrasion	Substrate metallic shavings, polymeric micro-debris
	Chemically Incompatible Materials	Processing fluid interactions causing matrix degradation and breakdown	Degraded elastomeric seals, plasticizing matrix residues
	Non-Dedicated Multi-Product Systems	Insufficient line clearance and cross-contamination from adjacent campaigns	Carryover particulate residues from preceding batches
	Poorly Designed Seals & Gaskets	Geometric gaps and structural mismatches causing physical material shedding	Fractured rubber fragments, gasket abrasive wear particles
	Inadequate Interconnection Sealing	Atmospheric pressure drops allowing external ingress or product egress	Extruded gasket fibers, environmental airborne dust
	Excessive Frictional Interfaces	Unmitigated high-friction contact zones driving fast mechanical wear	Pyrophoric metal dust, structural polymer wear debris
	Inadequate Filtration Mechanics	Pore size mismatch or bypass channels allowing downstream particle escape	Unretained process dust, structural filter fiber shedding
	Loose or Mismatched Components	Resonance-induced structural vibration leading to fastening interface wear	Metallic bolt shavings, fragmented fastener debris
	Decomposing & Aged Lubricants	Tribological breakdown and thermal cracking under high operational stress	Aggregated oil residues, degraded gel/lubricant particles
	Overheating Operational Surfaces	Hot spots causing rapid localized thermal degradation and charring	Carbonized process residues, burnt polymer flakes

**Table 4:** Mechanical and Operational Generation Sources.

Risk	Source/Unit Operation	Mechanism of Generation	Examples
**Process**	Sieving/Milling	High-velocity particle impact and mechanical stress against sizing screens	Fine powder, metallic screen fragments
	Mixing/Blending	Friction between particles and blender wall/impeller surfaces	Powder fines, fragmented granules
	Granulation	Mechanical shear and particle breakage during wet or dry processing	Granule fragments, airborne dust particles
	Drying	Fluidization forces shedding dried material or crystallized residues	Powder residues, crystalline particles
	Compression	Mechanical wear and friction of punches and dies during tablet formation	Metallic fragments, tooling shards, tablet fines
	Coating	Attrition, flaking, or shedding of solidifying coating polymers	Coating flakes, overspray residues
	Filling	Aggressive dosing mechanics generating particulates during container filling	Spilled powder, airborne fines
	Packaging	Shedding of particles during material transfer or sealing	Packaging film fibers, polymer fragments, dust
**Equipment with Moving Parts**	Bearings, Shafts, & Drives	Adhesive wear and friction between rotating internal components	Microscopic metallic shavings, polymeric debris
	Conveyors & Transport Belts	Mechanical abrasion and static attraction of belts against pulleys	Elastomeric belt fibers, fragmented product material
	Mixers & Agitators	Close-tolerance contact between impeller blades and vessel walls	Metallurgical wear particles, localized powder fines
	Pumps & Liquid Dosers	Frictional wear of internal impellers, rotors, or mechanical seals	Elastomeric seal fragments, microscopic metal filings
	Compressors & Pneumatics	High-pressure mechanical degradation and seal breakdown	Trace metallic particles, vaporized oil residues
	Tablet Tooling (Punches/Dies)	High-compression friction between punch tips, die walls, and formulation	Hardened steel fragments, black lubricant residues
	Filling Needles & Nozzles	High-speed, repetitive friction of reciprocating dosing parts	Delaminated plastic particles, metallic micro-shards
	Granulator Rotors & Mills	High-impact mechanical shear and blade attrition against raw material	Sub-micron granule fines, blade edge fragments
	Fluid Bed/Rotary Dryers	Aerodynamic shear or tumbling friction causing surface attrition	Friable dust particles, edge-sheared material fines
	Sealing Jaws & Wrapping Feeders	Repetitive mechanical crimping, cutting, and film-tension friction	Aluminum foil slivers, polymer film fragments
**Equipment with Non-Moving Parts**	Storage & Holding Tanks	Passivation layer breakdown, localized pitting, or fluid sedimentation	Ferric oxide scales (rust), surface flakes, settled particulate matter
	Transfer Pipelines & Ducts	Chemical oxidation, fluid-velocity erosion, or product residue baking	Rouging particles, process scale, baked-on residual deposits
	Process Filters & Sizing Sieves	Hydraulic pressure spikes, media blinding, or structural fiber shedding	Polymeric/glass filter fibers, trapped particulate clusters
	Static Seals & Gaskets	Elastomeric extrusion, thermal compression set, or chemical degradation	Degraded rubber fragments, elastomeric micro-debris
	Vessel Liners & Coatings	Thermal cycling stress or chemical exposure causing coating delamination	Polymeric liner flakes, epoxy chips, passivated metal slivers
	Material Handling Hoppers	High-velocity gravity impact and frictional abrasion during product loading	Substrate metallic particles, impact-sheared powder fines
	Structural Supports & Frames	Environmental humidity, chemical washdown exposure, or atmospheric oxidation	Structural rust fragments, delaminated paint chips, ambient dust

**Table 5:** Processing Aids, Cleaning Systems, and Packaging Vectors.

Risk	Source	Mechanism of Generation	Examples
**Processing Aids**	Improperly Selected Manual Tools	Frictional contact and structural wear of low-grade scoops or spatulas	Polymeric fragments, delaminated surface coatings
	Flexible Tools with Weak Structural Integrity	Structural fatigue, bending stress, and deformation causing fragmentation	Fractured plastic shards, sheared metallic slivers
	Incompatible Material Composition	Chemical leaching or matrix breakdown caused by product or solvent contact	Decomposed plastic matrix, corroded metallic particles
	Electrostatically Charged Aids	Triboelectric charging driving particle attraction and flash discharge	Electrostatic dust agglomerations, attracted ambient fibers
	Rough or Uncoated Tool Surfaces	High surface micro-roughness trapping, concentrating, and shedding fines	Abraded coating particles, trapped process dust
	Non-Dedicated Material Utensils	Inadequate line clearance causing multi-product carryover contamination	Residual particulate cross-contaminants from past batches
	Inadequately Cleaned Utensils	Poor sanitation validation causing product or surfactant baking	Dried process residues, crystallized detergent particles
	Overused Disposable Aids	Single-use material exhaustion leading to matrix tearing and shedding	Cellulosic paper fibers, frayed polymer fragments
	Poorly Maintained Tools	Macro-surface degradation from aggressive mechanical scrubbing or dropping	Deep-groove scratch debris, embedded contaminants
	Improper Tool Storage	Suboptimal storage exposing uncontained tools to ambient fall-out	Settled atmospheric dust, airborne environmental particles
**Cleaning Systems**	Abrasive Cleaning Formulations	Substrate surface micro-gouging and erosion from high-particulate scouring agents	Metallic micro-shaving debris, polymer matrix fragments
	Aggressive Chemical Cleaners	Passivation layer destruction or chemical oxidation leading to galvanic corrosion	Ferric oxide scale (rust), chemical degradation residues
	Trapped Cleaning Surfactants	Incomplete rinsing leading to localized solution concentration and surface crystallization	Dried surfactant precipitates, crystallized chemical deposits.
	Fiber-Shedding Sanitation Tools	Mechanical tearing and abrasion of low-grade wipes, mops, or brushes against components	Cellulosic lint filaments, non-woven polymer microfibers
	Scratched or Pitted Substrates	Material dislodged from surfaces damaged by aggressive mechanical scrubbing	Delaminated stainless steel slivers, polymer scratch debris
	Multi-Product Cross-Contamination	Inadequate cleaning validation and line clearance leaving past product on surfaces	Powder residues carryover, foreign particles
	Incomplete Post-Wash Drying	Retained moisture evaporation pooling dissolved solids onto product contact faces	Mineral evaporative residues (evaporation rings), water-spot scale
	Incompatible Cleaning Steps	Out-of-sequence chemical mixing driving solution neutralization or precipitation	Insoluble salt precipitates, gelatinous chemical reaction complexes
	Non-Dedicated Sanitation Assets	Mechanical cross-transfer of dirt, grease, and grime from non-controlled process lines	Foreign process debris, cross-contamination particulate chains
**Primary Packaging Materials**	HDPE/PET Plastic Bottles	Surface abrasion from bottle-to-bottle frictional nesting or polymer degradation during sorting	Polymeric micro-particles, static-bound microplastics
	Glass Bottles	Frictional glass-to-glass contact on accumulation tables or mechanical heel-to-heel chipping	Glass lamellae flakes, micro-shards, amorphous silica fines
	Plastic Caps & Induction Liners	Thread friction during high-torque capping or thermal drying/peeling of foil-induction layers	Polymeric cap shavings, crystallized adhesive flakes, liner fibers
	Metal Caps & Aluminum Crimps	Metal-on-metal friction, thread-gallbox corrosion, or jaw crimping stress on bottle necks	Aluminum flakes, anodized steel fragments, oxidation scales
	Blister Pack Foils & PVC/PVDC Films	Mechanical die-cutting stress, micro-tearing, or pocket formation stretching during thermoforming	Delaminated aluminum flakes, polymer film fragments
	Overwrap & Plastic Films	Mechanical tearing, web-guide friction, or triboelectric charging driving particle draw	Polymeric micro-filaments, electrostatically bound dust
	Suboptimal Handling & Storage	Friction inside secondary shipping boxes or exposure to ambient environmental fallout before filling	Cardboard corrugated dust, atmospheric particulates.

**Table 6:** Personnel and Human Intervention Variables.

Risk	Source	Mechanism of Generation	Examples
**Personnel**	Skin Exfoliation	Natural epidermal shedding accelerated by friction from movement under garments	Squamous epithelial cells, keratin flakes
	Hair & Facial Dermal Surfaces	Structural hair fracture or follicle shedding passing through porous head coverings	Hair fragments, dandruff particulates, sebaceous scales
	Physiological Secretions	Perspiration, surface lipids, or respiratory exhalation bypassing basic masks during exertion	Evaporated sodium salt crystals, dried lipid matrix residues, biological aerosols
	Transient Respiratory Events	Forceful coughing, sneezing, or deep breathing expelling high-velocity droplets	Saliva droplet nuclei, dried mucosal debris, microbial vectors
	Cosmetics & Skincare Products	Flaking or dispersion of applied substances	Powder residues, dried lotion flakes
	Plant-Floor Gowning	Flex-fatigue, friction, and fiber breakdown of non-woven or linting laundry fabrics	Cellulosic fiber lint, synthetic monofilament gowning fragments
	Protective Gloves	Elastomeric degradation, micro-tearing, or abrasive wear against sharp edges	Nitrile fragments, latex micro-shards, donning powder residues
	Suboptimal Gowning Discipline	Incorrect overlapping at sleeve/boot interfaces creating venting points during movement.	Exposed skin micro-flakes, macro-fibers from undergarments
	Aggressive Material Handling	Rough manual handling causing glove abrasion	Transferred glove polymer residues, localized skin oils
	Kinesic Plant Traffic	“Bellows effect” where rapid walking forces internal gown air through seams and cuffs	Expelled internal suit air, re-entrained ambient dust
	High-Velocity Foot Traffic	Turbulent air eddies from fast walking agitating and re-suspending settled floor powder	Re-entrained powder fines, dislodged floor coating particles

**Table 7:** Risks Associated with Particulate Matter in Oral Dosage Forms.

Risk—Probable Impact	
Oral Solids	Oral Liquids
Reduced Wettability—Hydrophobic particulates can coat the tablet surface, reducing water penetration and delaying disintegration.	Microbial Growth—Particulates can serve as a substrate or carrier surface for microbial proliferation in fluid environments.
Pore Blockage—External particles may clog tablet microstructures, hindering fluid absorption and slowing dissolution.	Filtration Difficulties—Suspended particulates can clog processing filters during manufacturing, leading to batch contamination or compounding errors.
Disintegration Disruption—Particulates may interfere with binder or disintegrant function, leading to incomplete tablet breakdown.	Leachables & Toxicity—Leachables and extractables, leading to drug degradation, toxicity, or immune reactions. Microplastics or rubber particles, causing irritation, toxicity, or bioaccumulation.
Altered Drug Release—In modified-release formulations, particulates can disrupt polymer swelling or gel formation, causing inconsistent drug release.	-
Physical Injury—Particulates may cause mucosal abrasions, esophageal injury, or GI perforation [7].	
GI Irritation—Particulates can lead to localized inflammation, discomfort, or exacerbate pre-existing GI conditions [10].	
Impaired Drug Absorption—Particulates can temporarily block or alter absorption sites, reducing drug bioavailability.	
Chemical Instability—Particulates may catalyze chemical degradation of Active Pharmaceutical Ingredient (API), reducing its potency.	
Drug Degradation—Increased formation of degradation by-products may pose toxicity risks and affect product safety.	

**Table 8:** Mitigation Strategies for Particulate Matter Contamination.

Particulate	Mitigation Strategies
Particulate contamination from raw materials (APIs & excipients)	Establish strong supplier qualification programs [11] Implement incoming material testing and screening [12] Store and handle materials under controlled conditions [13] Filter bulk liquids using ≤50-micron filters Screen solid materials before processing
Environmental particulates due to poor facility design and controls	Use non-shedding, durable facility materials [14] Maintain airlocks, pressurization, and cleanroom conditions [15] Minimize open handling of materials [11] Implement environmental monitoring programs [16]
Equipment wear generating metal or lubricant-based particulates	Conduct routine maintenance and inspections [17] Use wear-resistant materials for moving parts [17] Install metal detectors [17] and in-line filtration [8] Optimize lubrication practices to prevent excess residues [17]
Degradation of static equipment components (gaskets, filters, seals)	Implement scheduled replacement of components Perform regular inspections and validation of filter integrity Use low-shedding materials for seals and gaskets Conduct in-process screening of liquid dosage forms [17]
Processing aids shedding particles into formulations	Select chemically resistant, non-shedding tools Perform compatibility testing with drug substances Monitor processing tools for signs of wear Filter liquid dosage forms before final filling
Residues or particulates introduced from cleaning agents and abrasives	Use low-residue, non-abrasive cleaning agents Ensure thorough rinsing and filtration post-cleaning Avoid harsh scrubbing methods that degrade equipment Validate cleaning protocols to remove residual particles
Shedding or particulate leaching from packaging components	Source low-shedding, high-quality packaging materials Conduct extractable and leachable studies Implement stringent storage and handling controls Perform pre-use inspections of packaging materials
Human-generated particulates (hair, fibers, skin cells)	Enforce strict gowning and hygiene protocols [17] Train personnel in contamination prevention measures [18] Implement hygiene monitoring programs
Utilities introducing airborne or waterborne particulates	Maintain HEPA filters in HVAC and compressed air systems [19] Install filtration and screening at key utility points Regularly test purified water, air, and steam for particulates Conduct preventive maintenance on utility systems [19]

**Table 9:** Mechanisms of particulate formation and physical instability in APIs during manufacturing and formulation.

API Category	Primary Source/Mechanism	Impact on CQAs
Crystalline	Rapid solvent evaporation; high-shear crystallization milling	Crystal fracture or secondary nucleation alters particle size distribution, compromising content uniformity and dissolution kinetics.
Thermolabile & Hygroscopic	Solid-state thermal degradation; moisture-induced hydrolytic pathways	Formation of degraded particulate complexes, localized discoloration, and potentially toxic degradants.
Low-Solubility	Hydrophilic bridging during wet granulation; high-pressure compaction	Particle agglomeration compromises blend homogeneity, inducing erratic and incomplete drug release profiles.
High-Energy Processing	Mechanical stress during micronization, jet milling, or high-shear blending	Polymorphic transitions or amorphous surface generation induces thermodynamic instability and subsequent uncontrolled crystal growth.
High-Melting Point	Sub-optimal thermal drying; localized frictional overheating	Solid-state recrystallization or localized thermal degradation forms hard particulate masses that compromise matrix uniformity.
Amorphous Solid Dispersions	Thermodynamic instability during hot-melt extrusion or spray-drying; moisture exposure	Phase separation and phase transformation back to the stable crystalline form, resulting in hard crystal particulate growth that severely impairs bioavailability.

**Table 10:** Mechanistic pathways of particulate anomalies and physical defects in pharmaceutical excipients.

Category	Examples of Excipients	Primary Source/Mechanism	Impact on CQAs
Cellulose Based	Microcrystalline Cellulose, Hypromellose	Thermal stress; residual lignocellulosic impurities [116]; incomplete polymer hydration [117]	Microcrystalline Cellulose undergoes localized carbonization forming black particulate masses [118]; Hypromellose forms highly viscous, undissolved gel lumps that disrupt blend uniformity [119].
Sugars and Polyols	Lactose Monohydrate, Mannitol, Sorbitol, Sucrose, Dextrose	Amorphous-to-crystalline phase transitions during spray-drying; moisture-induced capillary bridging	Lactose exhibits Maillard browning or solid-state agglomeration [120]; mannitol and sorbitol undergo moisture-mediated recrystallization [121]; sucrose and dextrose undergo localized caramelization [122].
Starches	Native Starch, Pregelatinized Starch, Modified Starch	High mechanical shear forces and compressive stresses during granulation/tableting	Structural fragmentation or premature partial gel formation reduces blend homogeneity; highly compacted starch hornified particles impair disintegration.
Lubricants & Glidants	Talc, Magnesium Stearate	Delamination of mineral layers; static-induced agglomeration due to poor storage environment	Form heavy hydrophobic aggregates that disrupt powder flow, delay tablet disintegration, and introduce extrinsic mineral particulates.
Calcium Phosphates	Dibasic Calcium Phosphate	Inadequate deaggregation milling; moisture-activated crystal bridging	Formation of highly abrasive, hard particulate agglomerates that cause severe tooling wear and compromise tablet mechanical integrity.
Silica Based	Colloidal Silicon Dioxide	Moisture adsorption inducing silanol-mediated condensation	Irreversible silanol aggregation reduces glidant functionality, generating micro-particulates that impair powder flowability.
Polymeric Matrix Formers	Polyethylene Glycol, Polyvinylpyrrolidone, Cross-Linked Polyvinylpyrrolidone, Carbomers	High-temperature aging; incomplete polymer chain swelling and hydration	Generation of highly dense macro-lumps or gel-cores that compromise dissolution; unswollen carbomer structures manifest as visible translucent particulates [123].
Oils and Waxes	Stearic Acid, Hydrogenated Oils, Glycerides	Uncontrolled thermal cooling; polymorphic transitions during cold-chain storage	Premature solidification or transition to high-melting polymorphic forms yields hard, waxy particulate masses that hinder consistent drug release [124].
Gums and Proteins	Guar Gum, Xanthan Gum, Carrageenan, Gelatin	Sub-optimal hydration kinetics; localized thermal cross-linking	Formation of unhydrated, highly viscous cores or insoluble cross-linked gelatin networks (“flocs”) that appear as visible particulate defects.

## Data Availability

No new data were created or analyzed in this study. Data sharing is not applicable to this article.

## Abbreviations

The following abbreviations are used in this manuscript:

AI	Artificial Intelligence
API	Active Pharmaceutical Ingredient
APIC	Active Pharmaceutical Ingredients Committee
AQL	Acceptable Quality Limits
CAPA	corrective and preventive actions
CMO	Contract Manufacturing Organization
CPG	Compliance Policy Guide
CQAs	Critical Quality Attributes
EDS	Energy-dispersive X-ray spectroscopy
EMA	European Medicines Agency
FDA	U.S. Food and Drug Administration
FTIR	Fourier-transform infrared spectroscopy
GAMP	Good Automated Manufacturing Practice
GI	Gastrointestinal
GMP	Good Manufacturing Practices
HEPA	High-Efficiency Particulate Air
HSI	Hyperspectral imaging
HVAC	Heating, Ventilation, and Air Conditioning
ICH	International Council for Harmonisation of Technical Requirements for Pharmaceuticals for Human Use
ICP-MS	Inductively Coupled Plasma Mass Spectrometry
IPEC	International Pharmaceutical Excipients Council
PAT	Process Analytical Technology
PDA	Parenteral Drug Association
PDE	Permitted Daily Exposure
PMDA	Pharmaceuticals and Medical Devices Agency
QRM	Quality Risk Management
SEM	Scanning electron microscopy
TUPP	Technically Unavoidable Particle Profile
UV	Ultraviolet
XRF	X-ray fluorescence
